# Determinantes sociales del parasitismo intestinal, la desnutrición y la anemia: revisión sistemática

**DOI:** 10.26633/RPSP.2017.143

**Published:** 2017-11-07

**Authors:** Jaiberth Antonio Cardona-Arias

**Affiliations:** 1 Universidad de Antioquia Universidad de Antioquia Medellín Colombia Universidad de Antioquia. Medellín, Colombia.

**Keywords:** Determinantes sociales de la salud, parasitosis intestinales, desnutrición, anemia, revisión, literatura de revisión como asunto, Social determinants of health, intestinal diseases, malnutrition, anemia, review literature as topic, Determinantes sociais da saúde, enteropatias parasitárias, desnutrição, anemia, literatura de revisão como assunto

## Abstract

**Objetivo.:**

*Caracterizar las publicaciones relacionadas con los determinantes sociales del parasitismo intestinal, la desnutrición y la anemia en el ámbito mundial*.

**Métodos.:**

*Se realizó una revisión sistemática de la literatura científica en Pubmed, Science Direct, SciELO, LILACS y Google Scholar con ocho estrategias de búsqueda, garantizando exhaustividad y reproducibilidad en las fases de la guía PRISMA*.

**Resultados.:**

*Se incluyeron 18 estudios en malnutrición, 3 en parasitismo, 3 en anemia y 2 evaluaron simultáneamente parasitosis y desnutrición; 65,4% de Suramérica y 69,2% fueron realizados en niños. La prevalencia en parasitismo intestinal osciló entre 30,6% y 83,3%; en anemia de 19,7% a 48,0%; y en desnutrición de 0,0% a 67,8%. Se halló una mayor frecuencia de análisis de determinantes biológicos o psicosociales, los determinantes intermedios más estudiados se relacionan con la vivienda y los ingresos, y los estructurales fueron los menos investigados. Los determinantes sociales comunes a los tres eventos incluyen: vivir en hogares sin condiciones sanitarias, zona rural, características inadecuadas de la vivienda, provisión inadecuada de agua, barreras de acceso al sistema médico, bajo nivel educativo y edad de los padres, empleo precario y bajos ingresos*.

**Conclusión.:**

*La mayoría de publicaciones no desarrollan un análisis multinivel para los determinantes individuales, intermedios o estructurales. Se requieren mayores esfuerzos en políticas sanitarias relacionadas con el abordaje de los determinantes sociales de las desigualdades en parasitismo, desnutrición y anemia, principalmente en categorías como las políticas macroeconómicas, clase social, mercado de trabajo, cultura, valores y territorio*.

## Abstract (en)

***Objective*.:**

Characterize the publications on social determinants of intestinal parasitism, malnutrition, and anemia at the global level.

***Methods*.:**

A systematic review was conducted of the scientific literature in Pubmed, Science Direct, SciELO, LILACS, and Google Scholar with eight search strategies, guaranteeing completeness and replicability, following the phases of the PRISMA guidelines.

***Results*.:**

The review included 18 studies on malnutrition, three on parasitism, three on anemia, and two on both parasitism and malnutrition; 65.4% of the studies were from South America and 69.2% were carried out among children. The prevalence of intestinal parasitism ranged between 30.6% and 83.3%; anemia, 19.7% to 48.0%; and malnutrition, 0.0% to 67.8%. It was found that biological and psychosocial determinants were most frequently studied; the most frequently studied intermediate determinants were related to housing and income; and structural determinants were least investigated. The social determinants common to the three conditions include: living in homes with poor sanitary conditions, rural areas, inadequate housing, inadequate water supply, access barriers to the medical system, young parents with little schooling, precarious employment, and low income.

The majority of publications do not conduct a multilevel analysis for individual, intermediate, or structural determinants. Greater efforts are needed in health policies that address the social determinants of inequality with respect to parasitism, malnutrition, and anemia, mainly in categories as macroeconomic policy, social class, labor market, culture, values, and territory.

## RESUMEN

**Objetivo.:**

Descrever as publicações internacionais relacionadas aos determinantes sociais de parasitismo intestinal, desnutrição e anemia.

**Métodos.:**

Foi realizada uma revisão sistemática da literatura científica nas bases de dados PubMed, Science Direct, SciELO, LILACS e Google Scholar seguindo oito estratégias de busca para assegurar a completude e a reprodutibilidade nas fases do método PRISMA.

**Resultados.:**

Foram selecionados para a revisão 18 artigos sobre desnutrição, 3 sobre parasitismo intestinal, 3 sobre anemia e 2 sobre parasitismo e desnutrição. Destes estudos, 65,4% foram conduzidos na América do Sul e 69,2% em crianças. A prevalência de parasitismo intestinal variou entre 30,6% e 83,3%, de anemia entre 19,7% e 48,0% e de desnutrição entre 0 e 67,8%. A análise de determinantes biológicos ou psicossociais foi a mais frequentemente observada nos estudos, sendo os determinantes intermediários relacionados às condições de moradia e ao nível de renda os mais estudados e os determinantes estruturais os menos pesquisados. Entre os determinantes sociais comuns às três afecções destacam-se: viver em domicílios sem saneamento básico, viver na zona rural, condições inadequadas de moradia, abastecimento de água inadequado, barreiras de acesso aos serviços de saúde, baixa escolaridade, pais jovens, trabalho precário e baixo nível de renda.

**Conclusão.:**

A maioria de publicações não apresenta uma análise multinível dos determinantes individuais, intermediários ou estruturais. Faz-se necessário investir mais esforços em políticas de saúde direcionadas aos determinantes sociais das desigualdades em saúde relativas a parasitismo intestinal, desnutrição e anemia, sobretudo no que se refere a políticas macroeconômicas, condição social, mercado de trabalho, contexto cultural, valores e área geográfica.

Las parasitosis intestinales son infecciones causadas por diversos agentes etiológicos que pueden transmitirse por el consumo de agua o alimentos contaminados con materia fecal, penetración larvaria intradérmica desde el suelo, de persona a persona o de animales al hombre (1). En el ámbito mundial se estimaban cerca de 3,5 millones de personas infectadas con parásitos intestinales en el 2008 (2), constituyendo un problema para la salud pública por su elevada ocurrencia, riesgo de transmisión y endemicidad; sumado a su relación con condiciones geográficas, problemas de infraestructura sanitaria, vivir en zonas rurales, la pobreza, factores socioantropológicos de las comunidades, inadecuados hábitos higiénicos y baja escolaridad (3-6).

En términos clínicos estas infecciones generan pérdida del apetito, mala absorción intestinal, lesiones en la mucosa intestinal, anemia y desnutrición (7,8); esta última agudiza los problemas descritos debido a que una dieta deficiente en uno o varios nutrientes o una mala asimilación de los alimentos, aumenta la probabilidad de morir, disminuye el desarrollo cognitivo y puede generar daños irreversibles en el estado de salud (9-13).

En América Latina cerca de 53 millones de personas tienen un acceso insuficiente a los alimentos, por lo que la desnutrición constituye uno de los principales trazadores de las inequidades en salud en la región. Entre los factores que presentan una relación directa con su ocurrencia se encuentran la ingestión insuficiente de micronutrientes, la presencia de enfermedades infecciosas y los problemas en el cuidado infantil (12,13).

La relación entre el parasitismo intestinal y la desnutrición se agrava con la presencia de la anemia ferropénica, la cual presenta elevada frecuencia, afecta el desarrollo cognitivo y motor, disminuye el rendimiento escolar, retarda el logro educativo, y en general, impacta negativamente las condiciones socioeconómicas y de salud pública (14,15).

Lo expuesto evidencia el círculo vicioso del parasitismo intestinal, la desnutrición y la anemia, los cuales presentan nexos clínicos por sus efectos deletéreos sobre el desarrollo biofísico; epidemiológicos, dado que su presencia discurre en el mismo tipo de poblaciones y comparten múltiples factores de riesgo; socioeconómicos, debido a que afectan a la población más vulnerable y retardan el progreso de los países; y de salud pública en general, dado que constituyen trazadores de pobreza y desigualdades en salud (16,17).

No obstante lo expuesto, el número de publicaciones que estudian estos tres problemas de manera simultánea resulta escaso y en diversos escenarios no se aluden sus causas estructurales, muchas de ellas relacionadas con deficientes condiciones materiales de vida y aspectos sociohistóricos de las poblaciones afectadas. Frente a esta limitación surge el modelo de los determinantes sociales, el cual trasciende el análisis de los problemas de salud en un ámbito individual al incluir determinantes intermedios y estructurales que hacen explícita la relevancia de mejorar las condiciones de salud de la población, reducir las desigualdades, diseñar políticas acordes a las necesidades y posibilidades de acción de los diferentes niveles territoriales (municipal, departamental, e incluso nacional) e incluir el contexto socioeconómico y político de cada país como un factor explicativo de las inequidades en salud (16).

En este orden de ideas, los determinantes sociales son “*propiedades basadas en el estilo de vida afectadas por amplias fuerzas sociales, económicas y políticas que influyen la calidad de la salud personal*“, las cuales incluyen categorías como “*la enseñanza, el empleo, el nivel y distribución de los ingresos, la vivienda, el desarrollo infantil, la seguridad alimentaria, la nutrición, la raza, el género y el estrés*“ (17). La Organización Mundial de la Salud (OMS) los define como “*las circunstancias en que las personas nacen, crecen, viven, trabajan y envejecen*, determinadas por el tipo de políticas de los países y los patrones de distribución de los recursos materiales, el dinero y el poder; las cuales explican las desigualdades en salud entre países y al interior de estos (18). En términos operativos, la Organización Panamericana de la Salud (OPS) y la OMS analizan los determinantes sociales de la salud en tres niveles: el individual que incluiría aspectos psicosociales; conductuales y biológicos del sujeto; el intermedio referido a los recursos materiales, y el estructural que corresponde al contexto socioeconómico (18,19).

Este estudio tiene como objetivo caracterizar las publicaciones relacionadas con los determinantes sociales del parasitismo intestinal, la desnutrición y la anemia en el ámbito mundial.

## MÉTODOS

Se llevó a cabo una revisión sistemática de la literatura, siguiendo el siguiente esquema metodológico:

### Protocolo de búsqueda y selección de los estudios

Se aplicaron las fases de identificación, tamización, elección e inclusión de la guía PRISMA (*Preferred Reporting Items for Systematic Reviews and Meta-Analyses*) que se describen a continuación:

Para la identificación y búsqueda de las publicaciones se combinó el término “determinantes sociales“ con parasitismo intestinal, malnutrición, anemia y sus sinónimos, a través del operador booleano &; esto derivó en ocho estrategias de búsqueda: determinantes sociales AND anemia; parásitos, parasitismo, helmintos, helmintiasis, desnutrición, subnutrición y marasmo, con sus homólogos en inglés. En algunos contextos se emplean los términos parasitismo y parasitosis como sinónimos; sin embargo, los Descriptores en Ciencias de la Salud (DeCS) definen a las parasitosis intestinales como una enfermedad sintomática y por tanto se incluyó el término parasitismo en el protocolo de búsqueda como una estrategia para incrementar la exhaustividad en la selección de estudios. Este último término, asimismo, fue el más hallado en estudios de prevalencia.

La búsqueda se realizó en Pubmed, Science Direct, SciELO, LILACS y Google Scholar. Para garantizar la exhaustividad del protocolo, se realizó una búsqueda por especificidad para los términos incluidos en el tesauro DeCS, y por sensibilidad para los no incluidos en éste. A pesar de que el modelo teórico de esta revisión corresponde a los determinantes sociales de la salud de la OMS-OPS, también se aplicó el protocolo de búsqueda con el término “determinación social“ de la medicina social latinoamericana, sin hallar estudios adicionales.

Respecto a la tamización, con base en la lectura de los títulos y resúmenes de los manuscritos, se aplicaron los siguientes criterios de inclusión: a) tener los términos de búsqueda en título o resumen (en el caso de Google Scholar sólo aplica el filtro de título); b) publicaciones en seres humanos; c) ser un estudio original; y d) cuyo objetivo fuese el estudio de los determinantes sociales de al menos uno de los tres eventos.

En el proceso de elección se excluyeron los textos de citas de Google Scholar, los artículos que fueron retirados de las bases de datos o que no estaban disponibles; con base en la lectura del texto completo se excluyeron los manuscritos que no aludían los “determinantes sociales“ y en su lugar fueron clasificados por los autores como estudios de prevalencia, transversales, ecológicos, prospectivos o de casos y controles.

No se aplicaron restricciones de tiempo de manera retrospectiva, la búsqueda finalizó el 20 de febrero de 2017. Algunas sintaxis usadas fueron: en SciELO (ti:((ab:(determinantes sociales AND parásitos)))); en Pubmed (Social determinants[Title/Abstract]) AND malnutri tion [Title/Abstract], (Social determinants[Title/Abstract]) AND helminthiases [Title/Abstract]; en Science Direct TITLE-ABSTR-KEY(Social determinants) and TITLE-ABSTR-KEY(helminth); en LILACS tw:(determinantes sociales anemia) AND (instance:“regional“) AND ( db:(“LILACS“)) N = 1, y en Google Scholar allintitle: determinantes sociales anemia.

Se incluyeron los estudios que cumplieron las fases anteriores y fueron caracterizados con las variables: título y autores, año de publicación, país, enfermedad, población, prevalencia del evento, tipo de análisis realizado (cualitativo, bivariado o pruebas de hipótesis, multivariado o multinivel-jerárquico) y determinantes sociales incluidos en la publicación, éstos se agruparon en las categorías del modelo de la OMS-OPS de la siguiente manera: a) cuatro determinantes individuales: servicios de salud, factores psicosociales, conductuales y biológicos; b) cinco determinantes intermedios: entorno residencial, vivienda o situación material, ingresos o situación económica, trabajo doméstico y condiciones de trabajo; y c) nueve determinantes estructurales: clase social, género, edad, etnia, territorio, política macroeconómica, mercado de trabajo, políticas del Estado de Bienestar y cultura o valores.

### Reproducibilidad y evaluación de la calidad metodológica de los studios

Para la evaluación de la calidad de las publicaciones se aplicaron los 22 criterios de la guía STROBE (*Strengthening the Reporting of OBservational studies in Epidemiology*) calculando el porcentaje de estudios que cumplían cada uno de los ítems de la guía. Se garantizó la reproducibilidad en la búsqueda y la selección de las publicaciones aplicando el protocolo en dos ocasiones diferentes, con un intervalo de una semana, mientras que para la reproducibilidad de la extracción de la información se diseñó una base de datos en Excel la cual fue diligenciada en dos ocasiones diferentes para verificar la concordancia de los datos extraídos.

### Análisis de la información

Se realizó una síntesis cualitativa de las variables predefinidas en el protocolo de investigación. Posteriormente se estableció el porcentaje de estudios que incluyeron cada uno de los determinantes sociales de la salud del modelo de la OMS-OPS y se realizó una síntesis cualitativa de las variables que dan cuenta de los determinantes del parasitismo intestinal, la malnutrición y la anemia.

## RESULTADOS

En el cuadro 1 se presenta la frecuencia absoluta de estudios hallados con la aplicación de las ocho estrategias de búsqueda en las diferentes bases de datos consultadas. En este punto se debe indicar que la búsqueda con “determinación social“ restringido al título/resumen, no recuperó estudios en PubMed ni en Science-Direct, mientras que en SciELO, LILACS y Google Scholar esta búsqueda sólo generó cuatro estudios, los cuales se habían identificado con el término “determinantes sociales“.

**FIGURA 1.**
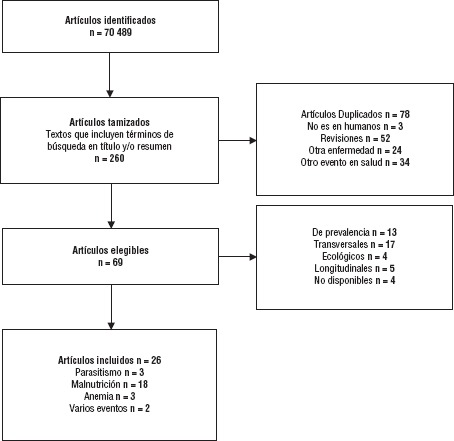
Flujograma de búsqueda y selección de los estudios

Se identificaron 70 489 publicaciones de las cuales sólo 260 incluían alguno de los términos de búsqueda en título o resumen, éstos se redujeron a 26 luego de aplicar los criterios de inclusión y exclusión, 18 en problemas nutricionales, 3 en parasitismo, 3 en anemia y 2 evaluaron simultáneamente los determinantes del parasitismo y la desnutrición (figura 1).

Los artículos se publicaron entre los años 2002 y 2017; 65,4% (n = 17) provenían de Suramérica, siendo Colombia el país con el mayor número de publicaciones (30,8%), seguido de Brasil (11,5%); la mayoría se desarrollaron en niños (69,2%), particularmente de la primera infancia. En relación con los tipos de estudio se hallaron dos cualitativos, uno de casos familiares y los demás correspondieron a investigaciones descriptivas, entre éstos el 56,5% eran estudios poblacionales o con muestreos probabilísticos y sólo tres realizaron análisis jerárquico o multinivel (cuadro 2).

**CUADRO 1.** Frecuencia absoluta de estudios identificados con la aplicación de las estrategias de búsqueda en las cinco bases de datos consultadas

Determinantes sociales^a^								
Estrategia de búsqueda	1	2	3	4	5	6	7	8
**Pubmed**								
Sin aplicar filtros	91	89	2	26	37	248	278	12
Aplicando restricción de búsqueda a la opción de Titulo/Resumen	6	10	1	6	1	48	14	0
**Science Direct**								
Sin aplicar filtros	5 588	3 333	727	715	135	6 366	1 869	196
Aplicando restricción de búsqueda a la opción de Titulo/Resumen/Palaba clave	7	16	0	7	0	36	11	0
**SciELO**								
Sin aplicar filtros	10	9	1	2	3	47	6	0
Aplicando restricción de búsqueda a la opción de Titulo/Resumen	8	6	1	1	2	36	6	0
**Google Scholar**								
Sin aplicar filtros	13 500	11 700	4 530	957	445	15 900	1 100	2 510
Aplicando restricción de búsqueda a la opción de Titulo	0	1	1	1	0	5	0	0
**LILACS**								
Sin aplicar filtros	4	5	4	2	2	33	3	4
Aplicando restricción de búsqueda a la opción de Titulo/Resumen	1	3	2	1	2	13	3	4

a 1 Anemia; 2 Parasites; 3 Parasitism; 4 Helminths; 5 Helminthiasis; 6 Desnutrition OR Malnutrition; 7 Undernutrition (OR Stunting OR Underweight OR Wasting), 8 Marasmo.

***Fuente:*** elaboración propia a partir de los resultados de la búsqueda.

***Fuente:*** elaboración propia a partir de los resultados de la búsqueda.

La mayoría de estudios reportó la medida de ocurrencia de la enfermedad; así, la prevalencia de parasitismo intestinal osciló entre 30,6% y 83,3%, algunos estudios reportaron la prevalencia por especie entre 0,0% y 45,3%; para la anemia fluctuó entre 19,7% y 48,0%, mientras que la desnutrición (aguda, crónica o global) estuvo entre 0,0% y 67,8% (cuadro 2).

**FIGURA 2.**
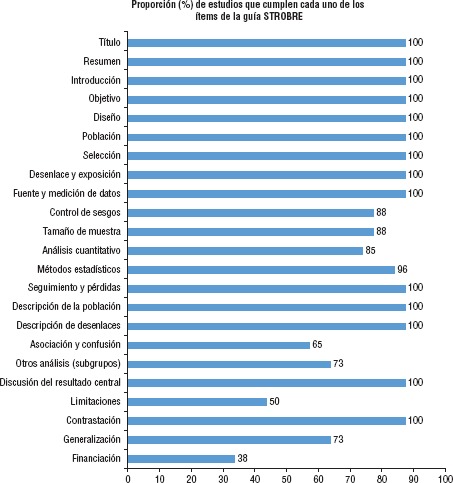
Evaluación de la calidad metodológica de los estudios según la guía STROBE

Los estudios incluidos presentaron buena calidad metodológica en la medida que se cumplieron entre el 70% (38) y el 100% (20,31,40,41,44) de los criterios de la guía STROBE, los ítems que no fueron aplicados en algunos estudios se relacionaron con la cuantificación o control de la confusión, hacer explícito el control de sesgos, el cálculo de tamaño de muestra, el análisis cuantitativo y de subgrupos, la validez externa y el declarar las limitaciones y el ente financiador de la investigación (figura 2).

**FIGURA 3.**
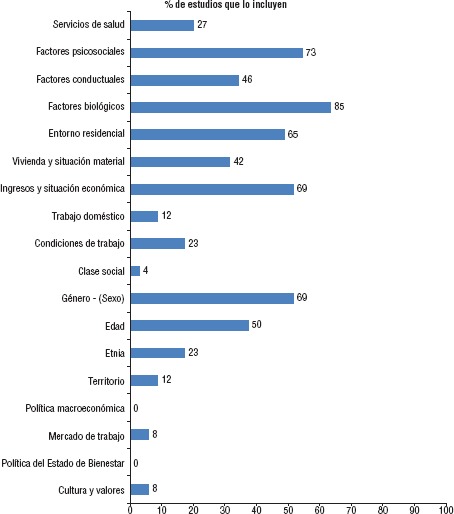
Distribución porcentual de los determinantes individuales, intermedios y estructurales descritos en los estudios

Con respecto a los determinantes sociales de la salud, las publicaciones se focalizan en aquellos del orden individual e intermedio, en los primeros la mayoría de artículos describieron al menos un aspecto biológico o psicosocial potencialmente relacionado con el parasitismo intestinal, la anemia o la desnutrición. En los determinantes intermedios los más estudiados se relacionan con características de la vivienda y el nivel de ingresos. Los determinantes estructurales fueron los menos estudiados, particularmente los relacionados con las políticas del Estado de bienestar, la política macroeconómica, la clase social, la cultura y el mercado de trabajo (figura 3).

En cuanto a parasitismo intestinal los determinantes incluyeron el vivir en hogares sin condiciones sanitarias o de zona rural, la presencia de características deficientes de la vivienda como tener el piso de tierra, no disponer de una adecuada provisión de agua y alcantarillado, el consumo de alimentos en la escuela, la presencia de barreras de acceso al sistema de salud, bajo nivel educativo de los padres, empleo precario, bajos ingresos y desnutrición (20-22,44).

En los problemas nutricionales los determinantes identificados fueron los problemas de lactancia materna (24), la baja edad y escolaridad de la madre (23-25,27,32,36,37,40,44), alto número de hijos (27,40), no recibir suplemento de vitamina A o hierro (28), no asistir a programas de control prenatal (32,40), no tener parto en centros de salud (32), habitar en zona rural (30), bajos ingresos del hogar (23,24,28,29,32,34,37), inadecuado acceso a agua (24,25), alto índice de Necesidad Básicas Insatisfechas o pobreza, empleo informal (26,36) e inequidad de ingreso (34). En los dos estudios cualitativos se describió la relevancia de la conciencia sobre los derechos de la sociedad civil, la movilización social, el énfasis en las políticas y acciones locales, la provisión de servicios públicos, el respeto a los valores locales y de género, el soporte social (33) y los procesos educativos como base para la construcción de capacidades multisectoriales a partir de la articulación de las políticas del gobierno, la asesoría de los tomadores de decisiones, la credibilidad de los resultados de investigación, la capacitación de los actores, y el fortalecimiento de la acción comunitaria (35).

Respecto a la anemia se documentó la asociación con la baja escolaridad de los padres, el consumo de agua no tratada (42), el estado nutricional, la edad de la gestante, el peso al nacer, la edad, el género, el inadecuado suministro de hierro (43), la pobreza, vivir en zona rural, no participar en programas de crecimiento y desarrollo, no estar afiliado a seguridad social en salud y una dieta deficiente (41).

## DISCUSIÓN

La mayoría de estudios se desarrollaron en población infantil lo que resulta coherente con algunas líneas de acción de la Comisión de Determinantes Sociales de la Salud dirigidas a esta población, las cuales enfatizan la necesidad de propender por equidad desde la niñez, garantizar el desarrollo de la primera infancia; promover la educación de calidad, los entornos saludables y el empleo digno para las familias; garantizar el acceso y uso de los servicios de salud; y mejorar las políticas de protección y seguridad social (18).

**CUADRO 2.** Caracterización de los estudios incluidos, según año, país, población, prevalencia del evento y modelo de análisis de los determinantes sociales de la salud (DSS)

Autor	Año	País	Población	Prevalencia %	Análisis DSS
**Parasitismo intestinal**					
Quintero K (20)	2012	Venezuela	3 388^a^ 3-60 años	*Ascaris* 3,73. *Trichuris* 1,13	Regresión logística
Garbossa G (21)	2013	Argentina	138 niños^a^	83,3	Pruebas de hipótesis y estratificado
Berrilli F (22)	2014	Costa de Marfil	306 niños 1-16 años de áreas rurales	21,6	Regresión
**Malnutrición**					
Marins V (23)	2002	Brasil	2 194 niños de 0-5 años^a^	1,3 a 9,1	Regresión logística
Ponce S (24)	2005	Ecuador	Poblacional, niños 0-5 años^b^	(NA)	Regresión lineal
Menegolla I (25)	2006	Brasil	1 283 niños 0-5 años	4,2 a 34,7	Regresión lineal
Gonzalez E (26)	2012	Colombia	Poblacional 0-5 años^b^	6,9	Regresión
Arias M (27)	2013	Colombia	169 niños 0-5 años	10,0 a 91,1	Pruebas de hipótesis
Deshmukh P (28)	2013	India	990 niños^a^	52,3%	Pruebas de hipótesis
Donini L (29)	2013	Italia	718 adultos mayores	26% mujeres y 16,3% hombres	Pruebas de hipótesis
Brcanski J (30)	2014	Serbia	3 347 niños 0-5 años^a^	8,7 a 72,9	Regresión logística
Carmona J (31)	2014	Colombia	200 familias y 46 niños 2,5-4 años	1,0 a 37,0	Pruebas de hipótesis
Mariños C (32)	2014	Perú	Niños 0-5 años^b^	(NA)	Regresión logística
Nandi S (33)	2014	India	17 entrevistas en profundidad y 10 grupales	NA	Cualitativo
Hanandita W (34)	2015	Indonesia	Poblacional^b^	NA	Regresión logística multinivel ^c^
Pridmore P (35)	2015	Chile y Kenia	Nutricionistas y enfermeras	NA	Cualitativo
Chatterjee K (36)	2016	India	1070 niños ^a^	54,3	Regresión
Man S (37)	2016	China	2 434 niños de 0-5 años	67,8	Regresión logística
Murcia M (38)	2016	Colombia	7 casos familiares	NA	Descripción de casos
Vallejo M (39)	2016	Colombia	239 niños ^a^ (117 indígenas)	1,6 a 43,6	Conglomerados
Huda T (40)	2017	Bangladesh	12 876 niños 0-5 años	36,0 a 43,0	Pruebas de hipótesis e índice de concentración
**Anemia**					
Vega R (41)	2008	Colombia	Poblacional de todas las edades^a^	26,5 a 48,0	Regresión e índice de concentración
Cotta R (42)	2011	Brasil	446 niños 0-7 años	22,6	Regresión jerárquica de Poisson ^c^
Falivene M (43)	2016	Argentina	483 niños 1-2 años	19,7	Regresión logística jerárquica ^c^
**Parasitismo y desnutrición**					
Alvarado B (44)	2006	Colombia	136 niños 1-2 años^b^	Infección 30,6 Desnutrición 12,5 a 2,9	Regresión logística
Carmona J (45)	2014	Colombia	1 600 menores de 15 años	Infección 0,0 a 45,3 Desnutrición 45,0 a 55,0	Pruebas de hipótesis

DSS: determinantes Sociales de la Salud. NA: No se aplica.

a Seleccionados aleatoriamente. ^b^ Censo. ^c^Análisis mutinivel.

***Fuente:*** elaboración propia a partir de datos publicados en los artículos incluidos en la revisión.

La relevancia de la población infantil radica en sus múltiples condiciones de vulnerabilidad física, mental y social; el constituir el grupo de mayor riesgo para los tres eventos analizados y el hecho que más del 30% de las muertes en esta población se relacionan con desnutrición. Se debe tener presente que la buena nutrición en esta etapa es determinante para garantizar el crecimiento y desarrollo, prevenir múltiples enfermedades, garantizar la buena salud física y mental; evitar infecciones (particularmente la diarrea de origen infeccioso); y otros daños irreversibles (46).

Es relevante hacer explícito que las inversiones en el desarrollo infantil presentan grandes ventajas clínicas, epidemiológicas, demográficas y de salud pública, las cuales redundan en mayor desarrollo social; entre ellas se destaca la reducción de costos sociales, el aumento de la probabilidad de tener éxito en la escuela y el mejoramiento de la salud individual y familiar. Invertir en los niños redunda en menores tasas de deserción escolar, de pobreza y de delincuencia; aumenta la probabilidad de terminación de la educación básica y de acceder a mejores trabajos en el futuro; disminuye las inversiones futuras que los gobiernos deben hacer en educación especial, rehabilitación, manejo de enfermedades, asistencia social y justicia penal, y potencia la productividad de las familias (47).

Estos aspectos evidencian la relevancia de investigar los determinantes sociales del parasitismo intestinal, la desnutrición y la anemia en cada país de la region como base para la planeación y gestión de acciones sanitarias e investigativas en estos tópicos. En este sentido, los resultados de esta revisión sistemática resultan de gran utilidad para investigadores, tomadores de decisiones, organizaciones comunitarias y demás actores formales e informales de las políticas públicas, al visibilizar la necesidad de ampliar el estudio biofísico o psicosocial de las enfermedades y trascender hacia la valoración de sus determinantes intermedios y estructurales como las características de la vivienda, la calidad de la infraestructura sanitaria, la educación de los padres, la ruralidad y la pobreza, en la medida que éstos constituyeron los determinantes de mayor relevancia para los tres problemas de salud pública analizados.

***Fuente:*** elaboración propia a partir de los resultados de la búsqueda.

Lo anterior es congruente con lo reportado para el caso latinoamericano, donde los determinantes sociales de la desnutrición incluyen el bajo nivel educativo, el desempleo, la zona de procedencia (mayor en el área rural), el género (mayor en mujeres) y la etnia (48). A esto se suman otros estudios que han demostrado cómo el aumento del nivel educativo y de ingresos reducen la probabilidad de desnutrición; mientras que la desigualdad de ingresos medida con el coeficiente de Gini aumenta la frecuencia de malnutrición (34).

En esta revisión se identificaron determinantes sociales comunes a los tres eventos sin desarrollar de manera exhaustiva un análisis jerárquico o multinivel. En este sentido, publicaciones previas han indicado que los estudios en este tópico no explican de manera exhaustiva las doctrinas que orientan sus investigaciones, la forma en que se establecen los procesos de determinación y, en general, se presenta un bajo desarrollo metodológico que ha limitado su avance y aplicación en la formulación de políticas en salud (49).

En términos metodológicos es pertinente indicar que los modelos multinivel analizan la relación entre los sujetos y el medio en que viven, separando el rol de las variables individuales y las comunitarias, con el fin de evitar sesgos cuando se analiza características de un nivel y se infieren conclusiones para otro, es decir, evitar la falacia ecológica y la atomista (50). En el caso de la investigación en determinantes sociales, el estudio de Falivene y cols. (43) analiza el problema de la anemia con variables jerarquizadas en cuatro niveles: a) en el proximal directo incluye la edad y el sexo; b) en el proximal indirecto agrupa características antropométricas y perinatales; c) en el intermedio - ambiental analiza prácticas de alimentación; y d) en el distal o de procesos sociales las Necesidad Básicas Insatisfechas, la cobertura médica y las intervenciones alimentarias. Otros estudios delimitan las jerarquías o multiniveles de forma diferente, como es el caso del estudio de Cotta y cols. (42) que en los aspectos proximales incluye la edad, la presencia de enfermedades, las medidas antropométricas y los antecedentes de anemia y parasitismo; en el intermedio toma el número de personas en el hogar, el consumo de agua no tratada, y en el nivel estructural la escolaridad de los padres y los ingresos (42,43).

Lo anterior evidencia una brecha importante entre la solidez teórica del modelo de determinantes sociales de la OMS-OPS y su limitado desarrollo metodológico para analizar el parasitismo intestinal, la malnutrición y la anemia, en tanto las publicaciones sistematizadas en este manuscrito aplican métodos de la epidemiología descriptiva o análisis jerárquicos que no resultan coherentes con los determinantes individuales, intermedios y estructurales. A esta diferencia en el desarrollo teórico y metodológico se suman limitaciones como el bajo número de estudios incluidos, probablemente relacionado con el hecho que la consolidación del modelo es relativamente reciente y el utilizar un conjunto limitado de términos de búsqueda tomados del tesauro DeCS. No obstante estas limitaciones, esta sistematización evidencia el interés de algunos investigadores por implementar el modelo teórico de la OMS-OPS, en problemas de alta repercusión clínica, sanitaria, económica y social.

***Fuente:*** elaboración propia a partir de los resultados de la búsqueda.

Concluyendo la mayoría de publicaciones se realizaron en América Latina, en población infantil, en los cuales se describen factores asociados con los tres eventos, como vivir en hogares sin condiciones sanitarias, zona rural, características de la vivienda, provisión inadecuada de agua, barreras de acceso al sistema médico, baja escolaridad de los padres, empleo precario y bajos ingresos; sin explicitar la tipología o análisis multinivel para los determinantes de orden individual, intermedio o estructural.

Con base en lo anterior, se recomienda mejorar el modelo teórico de la Comisión de los Determinantes Sociales de la Salud, con diseños metodológicos que evidencien la importancia del análisis multinivel y el respaldo empírico para cada país, principalmente en variables poco analizadas desde la investigación en parasitismo intestinal, desnutrición y anemia, como es el impacto de las políticas macroeconómicas de los países sobre estos problemas, la clase social, el mercado de trabajo, la cultura, los valores y el territorio; así como la captación de la heterogeneidad que se presenta en los demás componentes del modelo en cada localidad.

## Declaración.

Las opiniones expresadas en este manuscrito son responsabilidad del autor y no reflejan necesariamente los criterios ni la política de la *RPSP/PAJPH* y/o de la OPS.
